# Treprostinil inhibits proliferation and extracellular matrix deposition by fibroblasts through cAMP activation

**DOI:** 10.1038/s41598-018-19294-1

**Published:** 2018-01-18

**Authors:** Christopher Lambers, Michael Roth, Peter Jaksch, Gabriella Muraközy, Michael Tamm, Walter Klepetko, Bahil Ghanim, Feng Zhao

**Affiliations:** 1 Department of Thoracic Surgery, Lung Transplantation Program, Medical University Vienna, Währinger Gürtel 18-20, A-1090 Vienna, Austria; 2 Pulmonary Cell Research & Pneumology, Department Biomedicine, University & University Hospital Basel, Petersgraben 4, CH-4031 Basel, Switzerland; 3 Fourth Military Medical University, Xi’an, P. R. China

## Abstract

Idiopathic pulmonary fibrosis (IPF) is characterized by peripheral lung fibrosis and increased interstitial extracellular matrix (ECM) deposition. In IPF, tumor growth factor (TGF)-β1 which is the major stimulus of ECM deposition, and platelet derived growth factor (PDGF)-BB is a potent stimulus of fibrosis. Thus, the effect of Treprostinil on TGF-ß1 and PDGF-induced fibroblast proliferation and ECM deposition was investigated. Human peripheral lung fibroblasts of seven IPF patients and five lung donors were stimulated by PDGF, or TGF-β1, or the combination. Cells were pre-incubated (30 min) with either Treprostinil, forskolin, di-deoxyadenosine (DDA), or vehicle. Treprostinil time dependently activated cAMP thereby preventing PDGF-BB induced proliferation and TGF-β1 secretion. Cell counts indicated proliferation; α-smooth muscle actin (α-SMA) indicted differentiation, and collagen type-1 or fibronectin deposition remodeling. Myo-fibroblast indicating α-SMA expression was significantly reduced and its formation was altered by Treprostinil. Collagen type-I and fibronectin deposition were also reduced by Treprostinil. The effect of Treprostinil on collagen type-I deposition was cAMP sensitive as it was counteracted by DDA, while the effect on fibronectin was not cAMP mediated. Treprostinil antagonized the pro-fibrotic effects of both PDGF-BB and TGF-β1 in primary human lung fibroblasts. The data presented propose a therapeutic relevant anti-fibrotic effect of Treprostinil in IPF.

## Introduction

Idiopathic pulmonary fibrosis (IPF) is a rare, progressive and devastating interstitial lung disease resulting in a high mortality rate with limited treatment options^1,2^. IPF is mainly diagnosed in the elderly and is associated with specific histopathologic and/or radiological patterns of usual interstitial pneumonia (UIP)^2^.

Unfortunately, the pathogenesis of fibrotic diseases is not well characterized. Many fibrotic processes in the lung are associated with irregular wound healing, which leads to not well controlled proliferation and extracellular matrix (ECM) deposition^3,4^. In IPF, the main source for aberrant collagen type-I and fibronectin deposition are lung fibroblasts which are largely stimulated by TGF-ß1 signaling^4–6^. Beside TGF-β1, the growth factor PDGF-BB stimulates fibrotic processes and had been implicated as one of the driving factors in the pathogenesis of IPF, where both factors enhance each other’s function^4,7^. In line with this, the tyrosine kinase inhibitor nintedanib targeting the PDGF, VEGF and FGF signaling cascades induced an anti-fibrotic effect and counteracted TGF-ß induced ECM secretion in IPF^8^.

Recently, two new anti-fibrotic compounds, pirfenidone and nintedanib, were licensed for IPF treatment. Both drugs have a clinically documented efficacy on disease progression and target specific receptor linked protein kinases^9–11^. Nintedanib blocks the activation of three tyrosine kinase receptors: the platelet-derived growth factor-β (PDGF-β)-receptor, basic fibroblast growth factor and vascular endothelial growth factor^11,12^. Not much is known about the mechanism of pirfenidone, except it reduces fibroblast proliferation by reducing the action of p38 mitogen activated protein kinase (p38 MAPK)^13,14^.

Recently, the beneficial effect of prostacyclin analogues such as Treprostinil was demonstrated in patients with end-stage IPF and pulmonary arterial hypertension (PAH) by improving the right heart function without compromising systemic oxygenation^15^. Prostacyclins act through the IP receptor, which stimulates adenylate cyclase ensuing intracellular cyclic AMP (cAMP) generation^16^. The induction of cAMP by Treprostinil affected cell adhesion and differentiation of fibrocytes by downstream suppression of extracellular regulated kinase (Erk1/2 MAPK) signaling^17^. Prostacyclin analogues inhibited the proliferation of smooth muscle cells through Smad6 inhibition and activation of Smad1/5, suggesting a cross talk with TGF-β signaling^18^. In smooth muscle cells which expressed the prostaglandin EP-2 receptor, Treprostinil elevated cyclic AMP (cAMP), but had low activity on the other receptor types. Interestingly, these effects of Treprostinil were different from another prostaglandin analogue Iloprost^19^. In other conditions, cAMP formation modified the composition of the ECM through activation of the transcription factor cAMP response element binding protein (CREB), and thereby prevented the *de novo* deposition of collagen type –I, type- III and fibronectin^20^.

In the present study, the effects of Treprostinil on PDGF-BB and TGF-ß activated intracellular signaling were investigated in fibroblasts obtained from IPF patients and lung donors. Furthermore, the effect of Treprostinil on fibroblast remodeling and differentiation parameters, collagen type-I, fibronectin and α-SMA was assessed.

## Results

**Figure 1:**
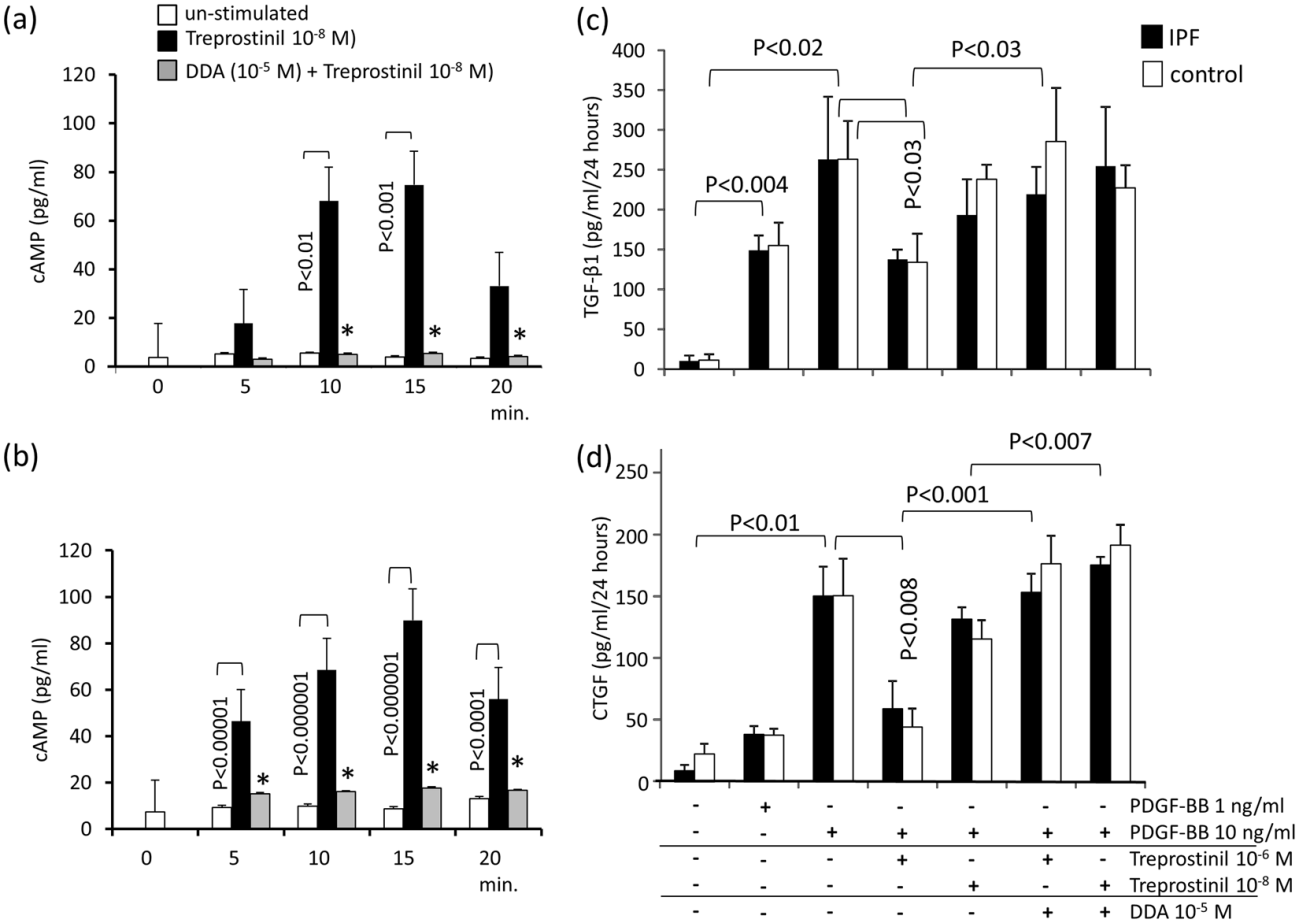
(**a**) Kinetic of cellular cAMP levels in control fibroblasts (n = 5) in the presence of Treprostinil or Treprostinil+ DDA. (**b**) Kinetic of cellular cAMP levels in IPF fibroblasts (n = 6) in the presence of Treprostinil or Treprostinil+ DDA. (**c**) Secretion of active TGF-β1 24 hrs after stimulation of fibroblasts with PDGF-BB and/or Treprostinil and/or DDA. Control fibroblasts (n = 5), IPF fibroblasts (n = 7). (**d**) Secretion of active CTGF 24 hrs after stimulation of fibroblasts with PDGF-BB and/or Treprostinil and/or DDA. Bars show mean ± S.E.M. of triplicated experiments. Statistics: Mann-Whitney U-test.

The level of intra-cellular cAMP did not change within 20 minutes in unstimulated control or IPF fibroblasts (Fig. 1a,b). Addition of Treprostinil 10^−8^ M significantly increased cAMP levels in both cell types and statistical analysis suggests that IPF fibroblasts generated more cAMP than control fibroblasts (Fig. 1b). Pre-incubation with DDA (10 μM) prevented cAMP increase in both cell types (Fig. 1a,b).

In order to understand the interactions of TGF-β1 and PDGF-BB, we next investigated the effect of PDGF-BB on the secretion of active TGF-β1 and of total connective tissue growth factor (CTGF), by the two fibroblast types. PDGF-BB increased the level of active TGF-β1 in fibroblast medium significantly over 24 hours (Fig. 1c) as well as that of CTGF (Fig. 1d). Neither the content of active TGF-β1 nor CTGF showed as disease specific increase comparing fibroblasts of IPF patients with cells of controls. DDA treatment counteracted the inhibitory effect of Treprostinil on PDGF-BB induced TGF-β1 activation and CTGF secretion suggesting a role of cAMP in its signaling action (Fig. 1c,d).

**Figure 2:**
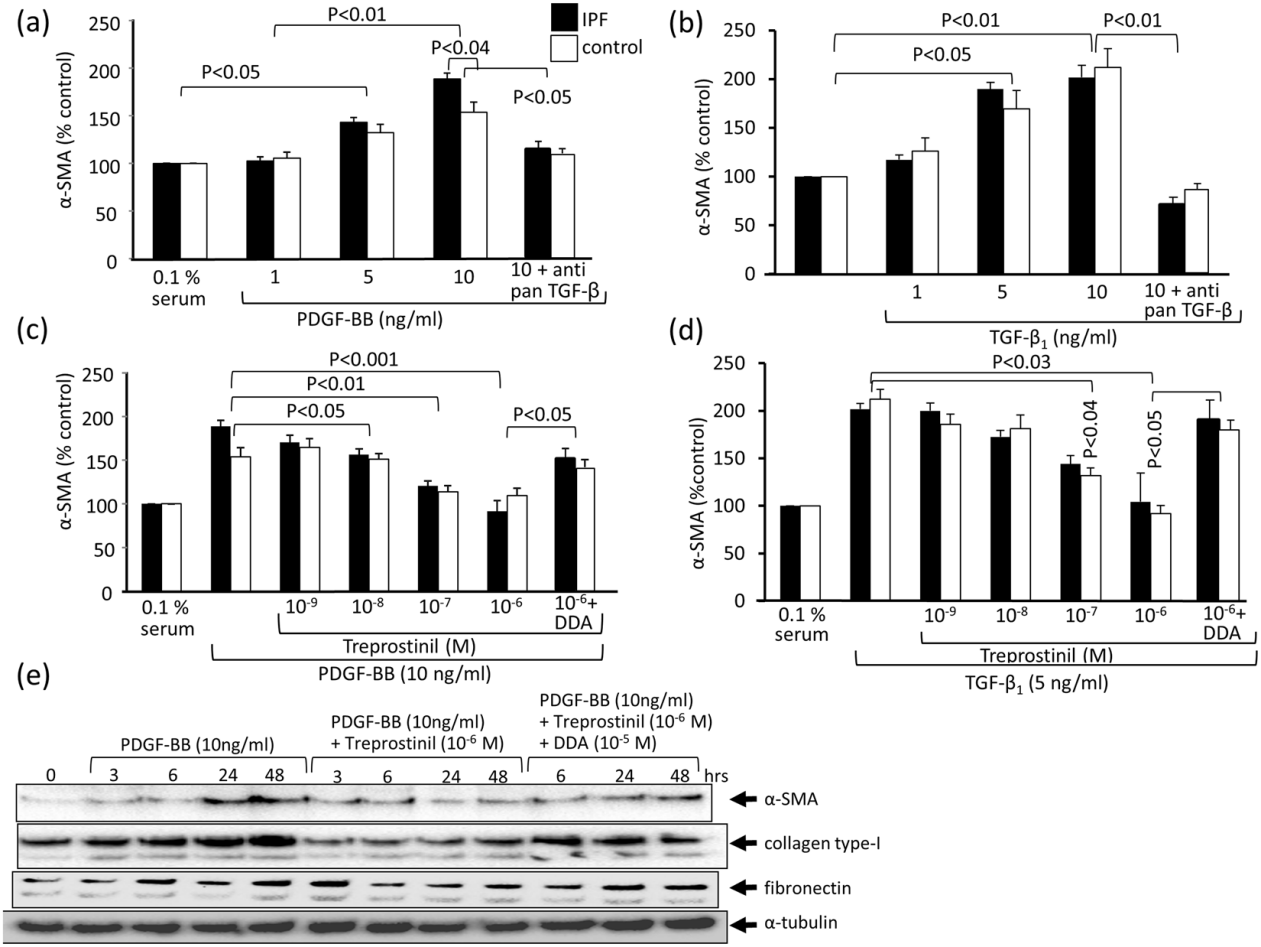
The effect of Treprostinil on PDGF-BB and TGF-β1 induced α-SMA expression. (**a**) Dose dependent stimulation of α-SMA expression by PDGF-BB in fibroblasts over 24 hrs as measured by immuno-blotting and densitometric analysis. Anti-pan TGF-β antibody (1 μg/ml) was added together with PDGF-BB. (**b**) Dose dependent stimulation of α-SMA expression by TGF-β1 in fibroblasts over 24 hrs as measured by immuno-blotting and densitometric analysis. Anti-panTGF-β antibody (1 μg/ml) was added together with PDGF-BB. (**c**) Dose dependent inhibition of PDGF-BB induced α-SMA secretion by Treprostinil; DDA (10 μM) was added 30 min before Treprostinil. (**d**) Dose dependent inhibition of TGF-β1 induced α-SMA secretion by Treprostinil; DDA (10 μM) was added 30 min before Treprostinil. Bars show the mean ± S.E.M. triplicates in all primary control and IPF cell lines. Statistics: Mann-Whitney U-test. (**e**) Representative immuno-blots for the effect of PDGF-BB (10 ng/ml), Treprostinil (10^−6^ M) and DDA (10^−5^ M) on the expression of α-SMA, collagen type-I and fibronectin (α-tubulin served as house keeping protein) after 42 hrs. Similar results were obtained in all other cell lines.

Under starving conditions α-SMA staining was very low, but was markedly increased after 24 hours in the presence of PDGF-BB (10 ng/ml), as well as by TGF-β1, suggesting a transposition of fibroblasts into myo-fibroblasts (Fig. 2a,b). In regard to fibroblast differentiation into pro-fibrotic myo-fibroblasts, we assessed the effect of neutralizing anti-pan TGF-β antibodies on α-SMA expression after 24 hours. As shown in Fig. 2a, PDGF-BB dose-dependently increased the expression of α-SMA in both fibrotic diseased and control fibroblasts, which was prevented by pre-incubation with neutralizing anti-pan TGF-β antibody. A similar stimulating effect on α-SMA was achieved by TGF-β1 stimulation, which was also abrogated by the presence of neutralizing anti-pan TGF-β antibody (Fig. 2b).

PDGF-BB induced α-SMA expression was dose dependently reduced when cells had been pre-treated with Treprostinil and the effect of the drug was counteracted by DDA (Fig. 2c). Similarly, Treprostinil dose dependently reduced TGF-β1 stimulated α-SMA expression, which was again counteracted by DDA (Fig. 2d). The results presented in Fig. 2c and d provide evidence that the action of Treprostinil on α-SMA expression is mediated by cAMP. A representative immuno-blot of the effects of PDGF-BB, treprostinil and DDA on the expression of α-SMA, collagen type-I and fibronectin is provided in Fig. 2e. We observed no significant disease specific difference on α-SMA stimulation or the action of Treprostinil.

**Figure 3:**
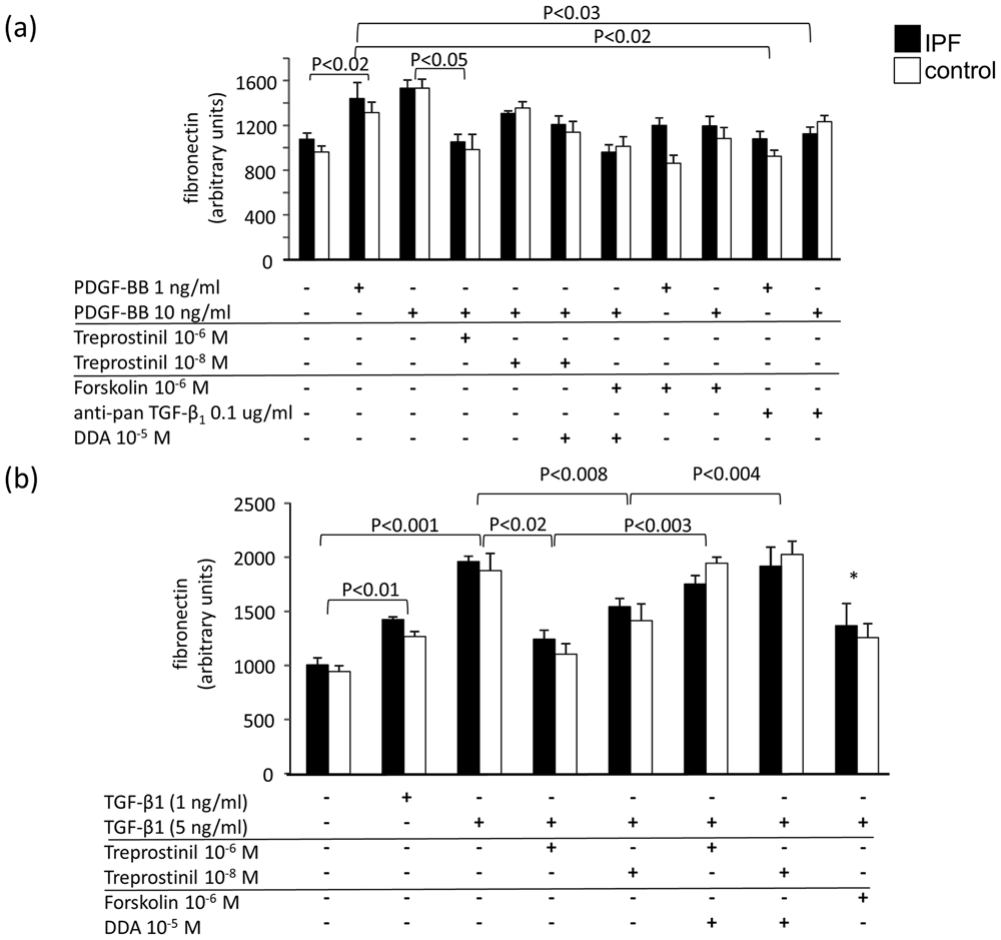
(**a**) Treprostinil inhibits PDGF-BB induced fibronectin deposition through TGF-β1 and cAMP. (**b**) Treprostinil inhibits TGF-β1 induced fibronectin deposition through cAMP signaling. Bars show the mean ± S.E.M. of triplicated experiments all primary control and IPF cell lines. Statistics: Mann-Whitney U-test.

We next analyzed the effect of Treprostinil on PDGF-BB induced ECM deposition. Unstimulated fibroblasts were characterized by cytosolic staining for fibronectin. In serum starved cells (24 hours) fibronectin was expressed on a basal level which was up-regulated by the presence of PDGF-BB within 24 hours (Figs 2e, 3a). When stimulated with PDGF-BB for 5 days we observed a significantly increased deposition of fibronectin (Fig. 3a). Treprostinil significantly reduced fibronectin deposition only at the highest concentration and this effect was not counteracted by DDA (Figs 2e, 3a). Interestingly, the presence of a neutralizing anti-pan-TGF-β antibody abrogated the stimulatory effect of PDGF-BB on fibronectin (Fig. 3a). The effect of TGF-β1 (5 ng/ml) on fibronectin deposition was significant at all concentrations after 24 hours (Fig. 3b). Pre-incubation with Treprostinil significantly reduced the stimulatory effect of TGF-β1 on fibronectin and this effect was interestingly in part sensitive to cAMP (Figs 2e, 3b). These results are in line with our earlier study reporting a target specific involvement of cAMP in the regulation of collagen type-I and fibronectin for cAMP stimulatory class of drugs, β2-agonists^20^.

**Figure 4:**
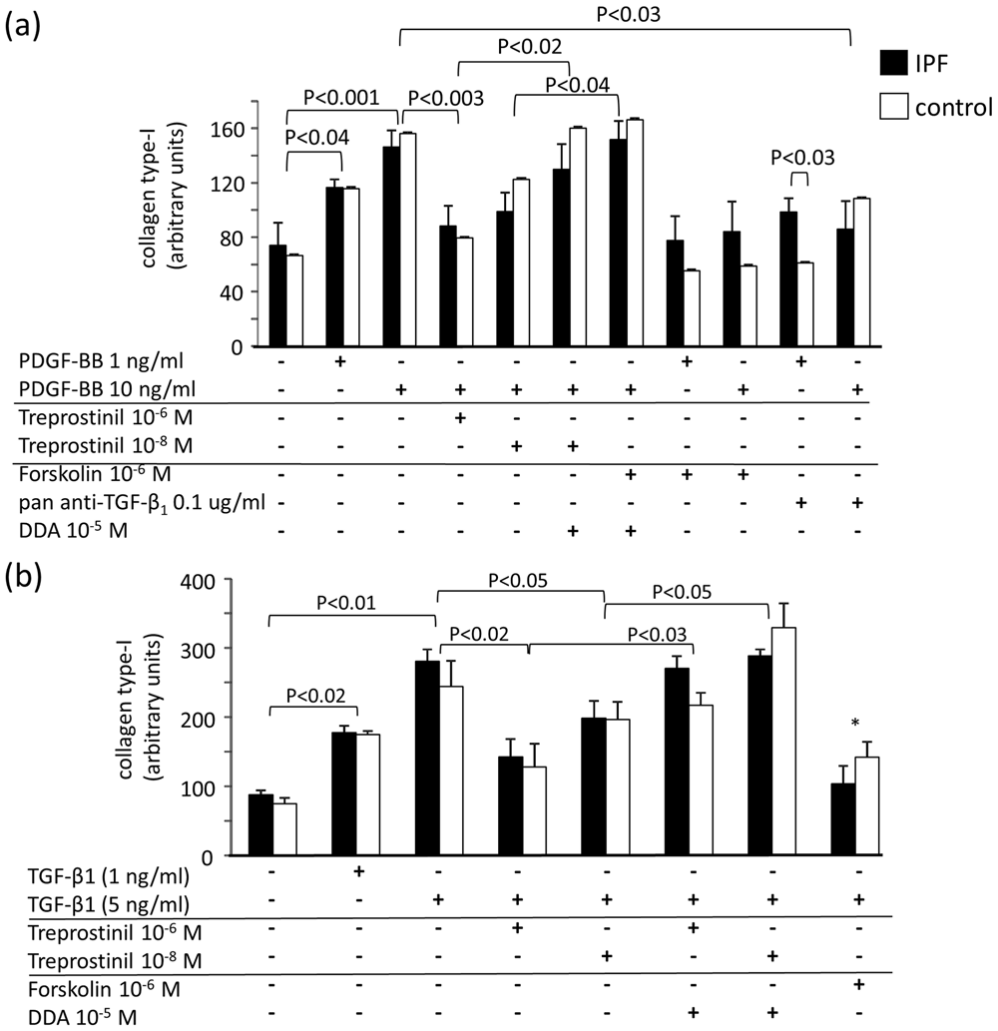
(**a**) Treprostinil inhibits PDGF-BB induced collagen type-I deposition through TGF-β1 and cAMP. (**b**) Treprostinil inhibits TGF-β1 induced collagen type-I deposition through cAMP signaling. Bars show the mean ± S.E.M. of triplicated experiments all primary control and IPF cell lines. Statistics: Mann-Whitney U-test.

PDGF-BB dose dependently increased collagen type-I deposition with no difference between control and IPF fibroblasts (Figs 2e, 4a). This effect was dose dependently reduced by Treprostinil through cAMP stimulation; since forskolin inhibited the effect of PDGF-BB and DDA inhibited the effect of Treprostinil (Figs 2e, 4a). In addition, anti-pan TGF-β antibody also significantly reduced the collagen type-I stimulation by PDGF-BB (Fig. 4a). This effect was significantly stronger in fibroblast of IPF patients. TGF-β1 also stimulated the deposition of collagen type-I dose dependently and Treprostinil prevented this effect (Fig. 4b). Since forskolin inhibited the collagen type-I deposition induced by PDGF-BB and DDA counteracted the effect of Treprostinil, cAMP plays a role in the signal transduction (Figs 2e, 4b).

Since PDGF-BB had no significant effect on other collagens (collagen-type-III, type-IV and type-VII, data not shown) we did not further investigate the effect of Treprostinil on these collagens.

**Figure 5:**
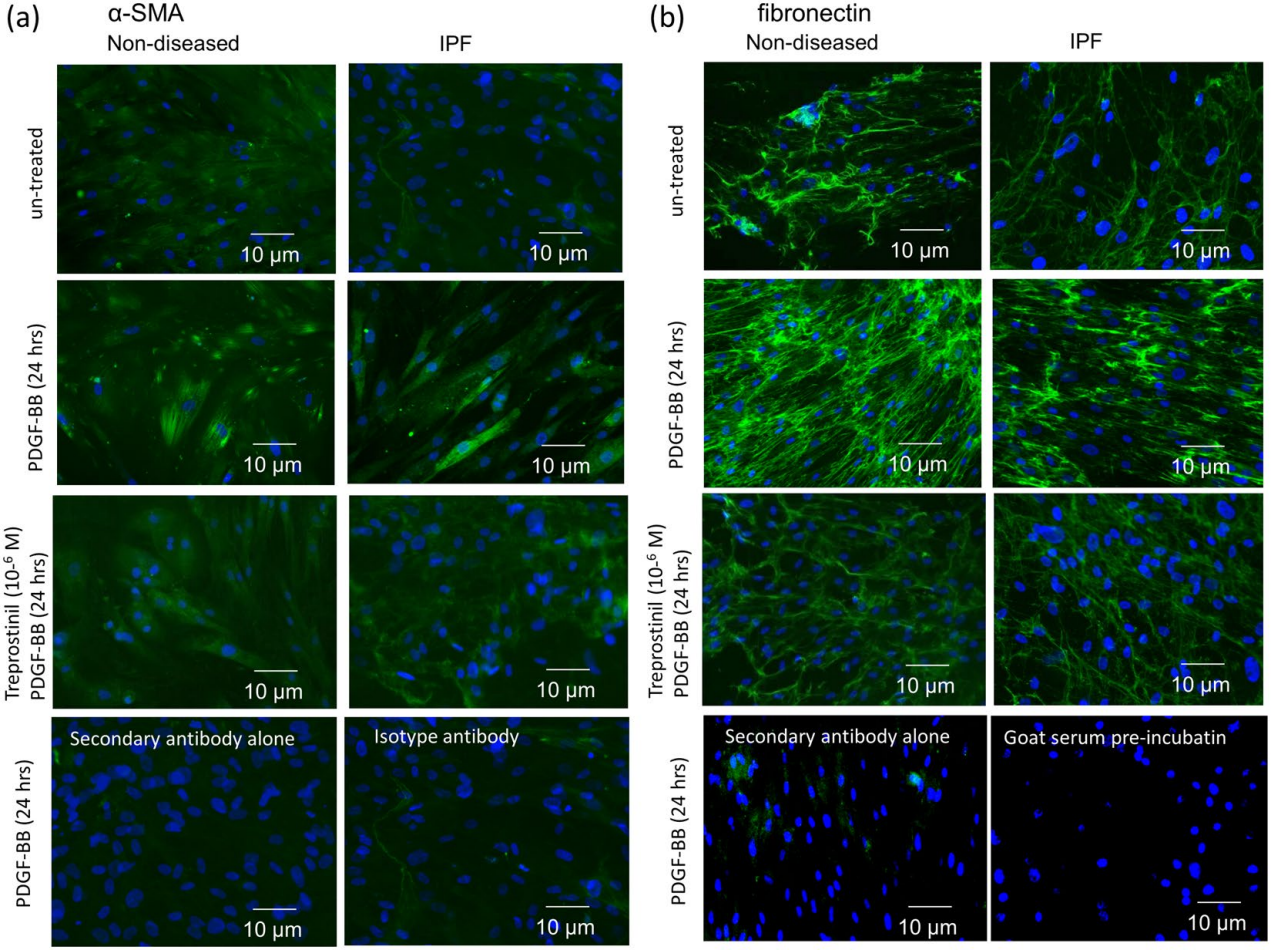
Treprostinil prevents α-SMA and fibronectin deposition and arrangement dose dependently. (**a**) PDGF-BB stimulated total synthesis as well as contractile fibril confirmation of α-SMA within 24 hrs and this was dose dependently reduced by Treprostinil. Fourth row shows images of cells incubated with the secondary antibody alone and with isotype antibodies of the first α-SMA specific antibody. (**b**) PDGF-BB induced fibronectin synthesis and network formation was dose dependently reduced by pre-incubation (30 min) with Treprostinil. Fourth row shows images of cells incubated with the secondary antibody alone and with isotype antibodies of the first fibronectin specific antibody. All pictures are representative for at least 5 additional experiments performed in independent primary cells.

**Figure 6:**
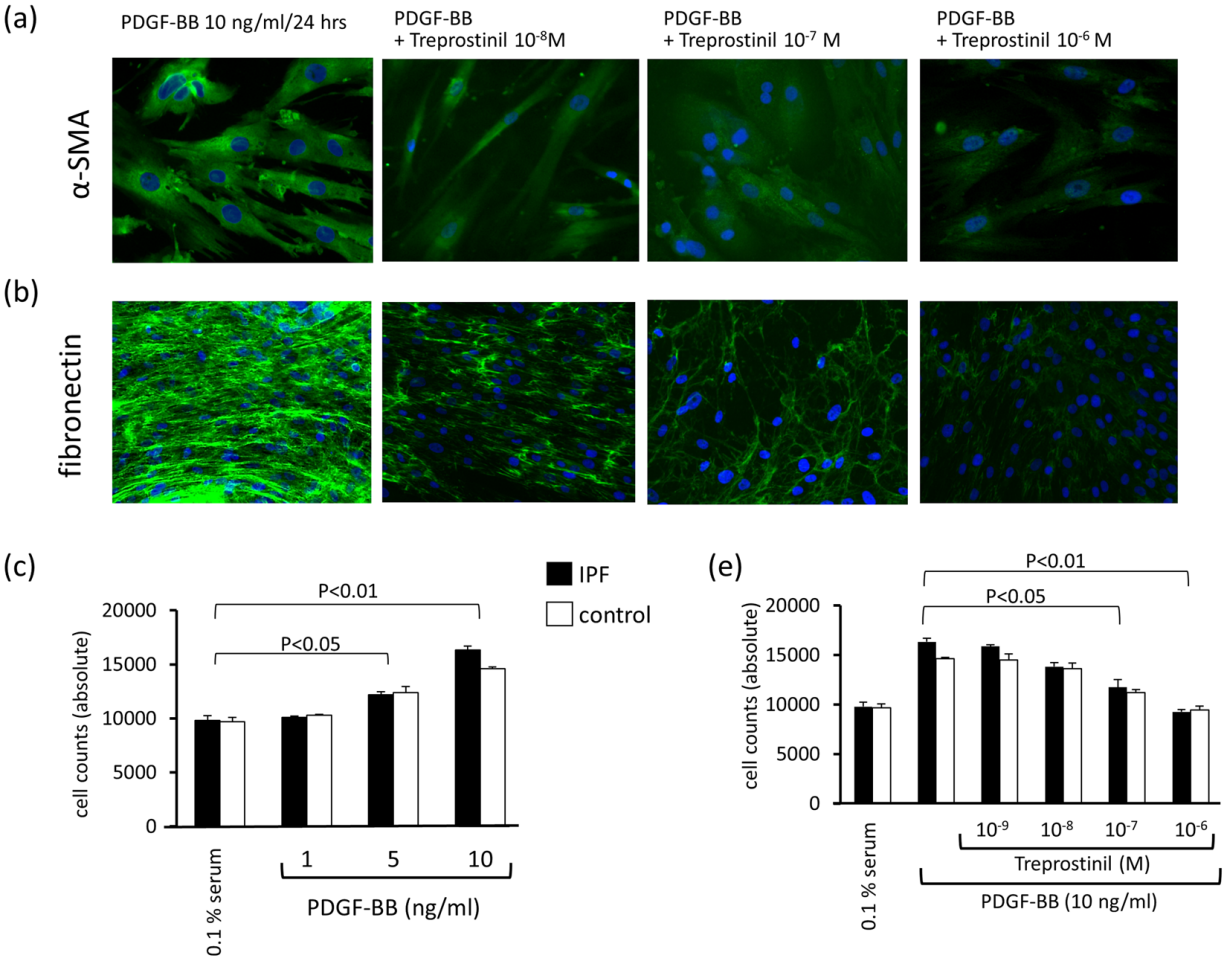
(**a**) Representative cyto-chemical staining (n = 3) for the dose-dependent reduction of PDGF-BB induced expression of α-SMA by Treprostinil at 48 hrs. (**b**) Representative cyto-chemical staining for the dose-dependent reduction of PDGF-BB induced expression of fibronectin (n = 3) by Treprostinil at 48 hrs. (**c**) Dose-dependent stimulation of fibroblast proliferation in three primary human non-fibrotic and three primary human IPF fibroblast lines (3 days). (**d**) Dose-dependent inhibitory effect of Treprostinil on PDGF-BB induced fibroblast proliferation in all primary control and IPF cell lines (3 days). Bars show the mean ± S.E.M. Statistics: Mann-Whitney U-test.

The function of α-SMA as well as of fibronectin depends on its arrangement within the cytosol or on the surface of the cells respectively. We have investigated the effect of Treprostinil on PDGF-BB induced α-SMA and fibronectin formation. As shown in Fig. 5a, resting fibroblasts expressed diffuse staining for α-SMA in the cytosol. After 24 hours of incubation with PDGF-BB, the formation of fibrilar α-SMA significantly increased in both non-disease and IPF derived fibroblasts (Fig. 5a, second row). Treprostinil prevented the PDGF-BB induced expression of α-SMA fibrils (Fig. 5a, third row). The fourth row of Fig. 5a depicts negative controls for the secondary antibody and an iso-type control antibody for the primary anti-α-SMA antibody. A representative immuno-fluorescence staining of the dose-dependent reduction of PDG-BB induced α-SMA expression and alignment by Treprostinil is depicted in Fig. 6a.

A similar effect of Treprostinil was observed in regard to the fibronectin network induced by PDGF-BB (Fig. 5b). Resting fibroblasts expressed a low level of fibronectin which was significantly up-regulated within 24 hours in the presence of PDGF-BB forming a cell surface, cell-cell linking network (Fig. 5b, second row). Treprostinil reduced the expression of fibronectin and changed its structure (Fig. 5b). The structure of fibronectin appeared more equally distributed and with reduced fibril formation in the presence of Treprostinil (Fig. 5b, third row). The fourth row of Fig. 5b depicts negative controls for the secondary antibody and an iso-type control antibody for the primary antibody. A representative immuno-fluorescence staining of the Treprostinil dose-dependent reduction of PDG-BB induced fibronectin expression is depicted in Fig. 6b.

Beside ECM composition, PDGF-BB also induced cell proliferation. Over a period of 3 days, PDGF-BB dose-dependently stimulated the proliferation of fibroblasts isolated from either patients with non-fibrotic lung diseases or from patients with IPF (Fig. 6c). We did not observe any significant difference of the proliferation rates comparing cells from IPF patients to that of healthy controls. Pre-incubation (30 minutes) with increasing concentrations of Treprostinil (10^−9^ to 10^−6^ M) dose-dependently reduced PDGF-BB-induced fibroblast proliferation (Fig. 6d).

## Discussion

In this study, we show that Treprostinil, a synthetic prostacyclin analogue can prevent PDGF-BB and TGF-β1 induced deposition of pro-fibrotic collagen type I and fibronectin, however, only collagen regulation involved cAMP. Therefore, Treprostinil may present a new therapeutic option for IPF.

IPF is a disease with limited therapeutic options and new strategies have to be evaluated. PDGF-BB and other pro-remodeling growth factors have been reported to be increased in IPF^7,21^. In IPF fibroblasts only TGF-β1 and peroxisome activity were up-regulated, while other remodeling parameters showed no IPF specific increase^22^. In contrast our data did not show any significant difference of TGF-β1 or CTGF secretion between fibroblasts from IPF patients and controls. This finding might be explained by the increased level of circulating growth factors in IPF patient serum resulting from organ specific pathology. In this context, we also did not observe any disease specific difference of fibroblast proliferation or synthesis of ECM components, as it was assumed on the basis of animal models^23,24^.

We observed that pre-incubation of fibroblasts with Treprostinil reduced proliferation without a disease specific effect. The anti-pro-fibrotic effect of Treprostinil on PDGF-BB on cell proliferation may involve cAMP, which is supported by our earlier study showing that long acting muscarinic receptor antagonists rescued the cAMP signal activated by long acting β2-agonists in human lung fibroblasts^25^. In contrast to our data, IPF models implied that cAMP plays a progressive role in fibrosis, however, this claim was only supported indirectly by the unspecific activation of transcription factors that are usually activated by cAMP^26,27^. Our study suggests that the activation of cAMP by Treprostinil overrules the stimulatory effect of TGF-β1 and/or PDGF-BB, while the details of the molecular biological level on which this signaling interference occurred remain unclear.

It has been shown that in IPF the metabolism of certain ECM components seems to be disturbed which is due to overly expressed TGF-β1^28^. In the context of this modified deposition of specific ECM components we reported that at least collagen type-I synthesis and deposition involves cAMP^20,29^. Thus, the increase of cAMP by any drug may reduce collagen type-I synthesis and deposition and therefore present a possible benefit for IPF therapy. Collagen type I seem to further promote the fibrotic process in IPF and also activate immune cells such as macrophages^30^. An increased deposition of collagen type I in isolated human IPF fibroblasts was related to malfunctioning peroxisomes^22^. An additional mechanism that increases collagen type I synthesis in IPF was the lack of micro-RNA 96 which increased FoxO3a^31^. The contributing role of collagen type I to the progression of the disease was supported by the observation that the expression of collagen type I binding proteins correlated with the severity of IPF and that their inhibition reduced fibrosis^32,33^.

Furthermore, the recently recognized drugs Nintedanib and Pirfenidone have been reported to down–regulate collagen type I synthesis and deposition^4,34^. Interestingly, there is a link of cAMP to Pirfenidone, since L-type calcium channels respond to Pirfenidone in a cAMP dependent mechanism^35^. Therefore, it might be hypothesized that the beneficial action of Pirfenidone may be achieved by a not well studied mechanism involving cAMP. Since two other IPF drugs, Pirfenidone and Nintedanib, have been shown to reduce the fibular arrangement of α-SMA in IPF fibroblasts and thereby reduced the generation of myo-fibroblasts transformation^36^ a similar action may be achieved by Treprostinil. Treprostinil had a similar effect as the other two drugs on the overall synthesis of α-SMA as well as it reduced the formation of contractile fibrils, suggesting a reduction of contractility, which may add to the beneficial effects of the drug.

Fibronectin expression was also increased in fibrotic lung tissues and this was reduced by Nintendanib and Pirfenidone, which resulted in reduced remodeling and fibrogenesis^37,38^. Treprostinil had a very similar effect on fibronectin synthesis and structure in both control and IPF fibroblasts. This finding suggests that Treprostinil may reduce mobility of fibroblasts and at the same time improving the re-establishment of epithelial cells^39^. In line with the observation of Ramos-Mondragon^35^, our study implicates that cAMP plays an important role in the anti-proliferative based therapy of IPF.

In a mouse model inflammation and remodeling of the ECM modulated prostacyclin induced cAMP activity which was suppressed by fibronectin through the activation of phosphodiesterase^40^. In platelets, cAMP levels were distinctly regulated by different prostanoid receptors, with IP1 and DP1 receptors being most effective, followed by EP4 and EP3 receptors^41^. Possible differences of EP2 and DP receptor dependent cAMP production in fibrotic diseases and the involvement of ECM components have not been investigated. Our data suggests that cAMP can be activated by prostacyclins and thereby may inhibit ECM deposition caused by fibrosis relevant growth factors including TGFβ or PDGF-BB.

Our results indicate that prostaglandin analogues may have a therapeutic potential in IPF, however, we only investigated a single compound and it has to be investigated if other prostaglandin analogues exert the same or similar anti-fibrotic effects. A second limitation of the study is that at this stage we cannot confirm the *in vitro* results in patients. Another limitation may be the lack of data confirming the drugs action in an animal model. However, we would like to point out that animal models do not fully reflect the human disease. Bleomycin is the most frequently used model for experimental lung fibrosis in animal, inducing patchy parenchymal inflammation, epithelium injury and myo-fibroblast formation; however, these pathologies do not resemble IPF^42^. As summarized by Degryse and Lawson, none of the available animal models for IPF (bleomycin, silica, fluorescein isothiocyanate, irradiation or transgenic vectors) fully reflects the human disease, most often lacking the pattern of interstitial pneumonia^43^. Therefore, we used human diseased primary cells, which often show much less variance than expected^11^.

In conclusion, our data suggest that Treprostinil may have a beneficial effect by preventing pro-fibrotic proliferation and matrix synthesis in IPF through cAMP. However, this new mechanism needs to be further investigated.

## Methods

All described experiments were performed in accordance with relevant guidelines and regulations.

### Tissue donors

Human primary lung fibroblasts were isolated from tissue explants obtained from seven patients with proven diagnosis of IPF and five healthy donor lungs. All tissue samples were obtained after written informed consent of each patient/donor and with the approval of the local ethical committee (AKH Vienna, Austria). All relevant patient data are displayed in Table 1.

**Table 1:** Clinical characteristics of IPF patients.

Patients	Age	gender	Treatment (lung)	FEV1 (% predicted)	HISTO (explant)	Smoking history Pack years (Year of smoking cessation)
#1	52	male	Spiriva, Revatio LTOT	FVC 2,24l (48,6%) FEV1 2,18l 49,3%)	UIP	20 PY (1998)
#2	51	female	Cortison	FVC 0,83l (31%) FEV1 0,58l (25%)	UIP + NSIP	NO
#3	48	male	Pirfenidon/Revatio	FVC 1,7l (35.02%) FEV1 1,57l (40%)	IPF + sPAH	NO
#4	46	female	Seretide, Combivent,Urbason, Pantoloc, Mucibene,	FVC 0,99l (29%) FEV1 0,72l (25%)	UIP	NO
#5	63	male	Aspirin 100	FVC 3,57l (74%) FEV1 2,76l (76%)	UIP	20PY (2005)
#6	65	male	Prednisolon, Pirfenidone	FVC 2,62l (61,7%) FEV1 2,44l (72,8%)	UIP	PY unknown (1990)
#7	40	female	Prednisolon,	FVC 0,94l (30,1%) FEV1 0,88l (32,2%)	UIP	unknown
Mean ± SEM	52.14 ± 3.6			FVC: 44.2 ± 6.3 FEV1:45.8 ± 10.1		

### Fibroblasts

Fibroblasts were isolated from tissues over 14 days, in cell type selective medium (CellnTec Advanced Cell Systems AG, Bern, Switzerland) as described earlier (Eickelberg *et al*., 1999). Afterwards, fibroblast were expanded in PRMI-1640 supplemented with 10% fetal calf serum, 20 mM HEPES, 8 mM L-glutamine (GlutaMAX), and 1x non-essential amino acid mixture (all: Gibco/BRL, Thermo Fisher Scientific, Switzerland). Cells were used between passages 3–6 and characterized by their long stretched spindle phenotype which stained positive for fibronectin and inducible staining for α-smooth muscle actin (α-SMA). A representative cyto-chemical staining is shown in Fig. 1a.

### Stimulation

Fibroblasts were grown to confluence and were pre-treated with Treprostinil at increasing concentration (10^−9^ to 10^−6^ M) for 30 minutes before being stimulated. TGF-β1 was used at different concentrations ranging from 0.1 to 5 ng/ml. PDGF-BB was used at a concentration of 10 ng/ml, which is the optimal concentration for proliferation by direct cell counts we defined earlier^8^.

### Cell proliferation

Fibroblast proliferation was determined by direct cell counts using a Neubaur chamber slide) at days 0, 3 and 5 in the presence and absence of PDGF-BB.

### TGF-β1 and Connective Tissue Growth Factor (CTGF) secretion

The content of TGF-β1 and CTGF in the cell culture medium was determined by commercial ELISAs as advised by the distributor (antikoerper-online.de, Aachen, Germany) in the cell culture medium of confluent cells collected at 24 and 48 hours after stimulation or drug incubation as described earlier^20^.

TGF-β1 ELISA were performed following the instruction of the distributor, except that the samples snap frozen after stimulation and were not activated by acid treatment, in order to only determine the content of active TGF-β1 and not that of total TGF-β1.

### Extracellular matrix deposition

The deposition of collagen type-I and -IV and of fibronectin was determined by a cell based in-house developed ELISA as described earlier^20^. All antibodies used for the ELISA were purchased from Santa Cruz Bio Technology, Santa Cruz, USA (COL1A1 SC-8784, COL4A1 SC-385020, fibronectin SC-6952, α-SMA SC-53015) and diluted in blocking buffer 1:100. Secondary antibodies were species specific for first antibodies and diluted 1:500 in blocking buffer, incubation was 1 hour at room temperature (Santa Cruz Bio Technology) and visualized for ELISA reader after 3x washes with PBS by a horse radish peroxidase (Thermofisher Scientific).

Deposition of α-SMA and fibronectin was also determined by immuno-cytochemistry in cells which were grown on cover slips and either stimulated with PDGF-BB alone or in combination with Treprostinil for 24 and 48 hours. Cells were then washed 1x with PBS, fixed for 2 × 5 minutes in 4% formalin, 1x washed with PBS containing 0.1% Triton-X100 for 15 min. while slowly shaking, blocked for 30 minutes (blocking buffer: PBS, 0.01% Tween 20, 2% bovine serum albumin), before being incubated overnight (4 °C) with the first antibody. After 3x washes with PBS, slides were incubated with a second FITC labelled antibody (30 minutes room temperature), nuclei were stained by DAPI and pictures were taken after 3x washes in PBS by microscope (EVOS FL cell imaging system; Thermofisher Scientific, Switzerland).

Iso-type control antibodies: mouse IgG iso-type control for α-SMA was sc-2339 (Santa Cruz Bio.) and for fibronectin iso-type control was performed by pre-incubating the first antibody in goat serum (cat# 16210064, ThermoFisher Scientific) for 30 minutes.

### Immuno-blotting

Total protein was collected from confluent fibroblast layers in RIPA-buffer [25 mM Tris (pH 7.4), 150 mM NaCl, 0.1% SDS, 0.5% sodium deoxycholate, 1% Triton X 100] and were size-fractionated as described earlier^20,29^ using t.

### Statistics

The data of the two fibroblast groups were compared by Student’s t-test (two-tailed, paired). The effect of the drug was tested by ANOVA or by Mann-Whitney U-test, as appropriate. A P value of <0.05 was considered as statistically significant.

### Data availability

All original data are available on request from the corresponding author.

## Electronic supplementary material

Supplementary Information
