# Supplementary material for: Treprostinil inhibits proliferation and extracellular matrix deposition by fibroblasts through cAMP activation
Source: Sci Rep. 2018 Jan 18;8:1087. doi: 10.1038/s41598-018-19294-1 (PMC5773699; doi:10.1038/s41598-018-19294-1)

Treprostinil inhibits proliferation and extracellular matrix deposition by fibroblasts through cAMP activation

*Christopher Lambers[1], Michael Roth[2], Peter Jaksch[1], Gabriella Muraközy[1], Michael Tamm[2], Walter Klepetko[1], Bahil Ghanim[1], Feng Zhao[2, 3]

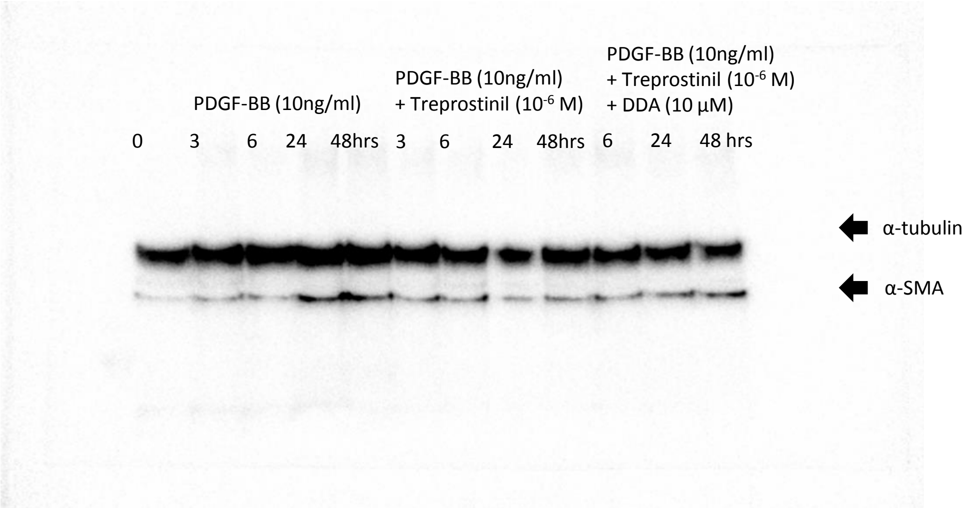

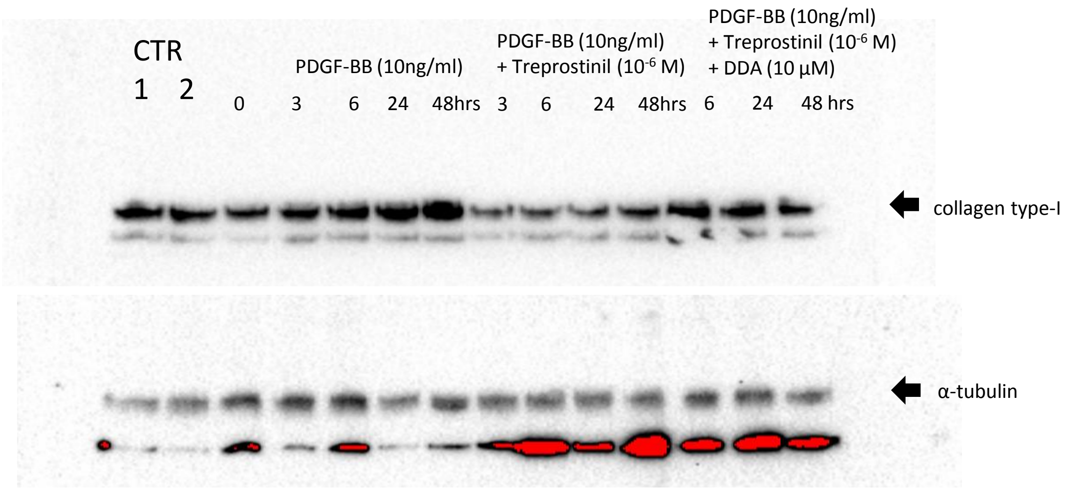

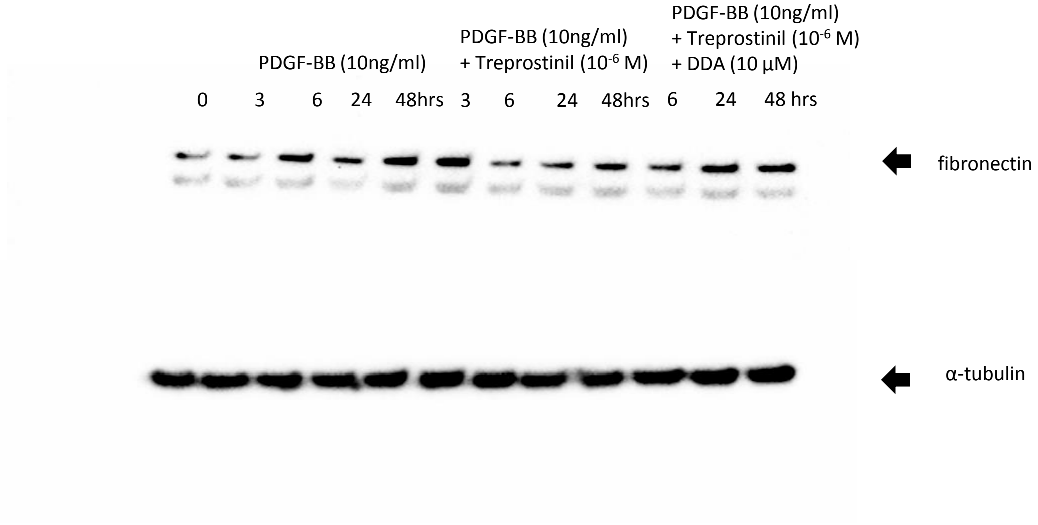

Supplement: Supplementary file 1 — Supplementary Information [file 41598_2018_19294_MOESM1_ESM.pdf]
